# Understanding nursing graduates’ intention to work with older adults: An exploratory structural framework

**DOI:** 10.1016/j.ijnsa.2026.100628

**Published:** 2026-07-12

**Authors:** Maria José Catalão, Helena Arco, Nuno Carrajola, João Tavares

**Affiliations:** a Department of Health Sciences and Technologies, School of Health, Portalegre Polytechnic University, Campus Politécnico 10, Portalegre, 7300-555, Portugal; b CARE-Research Center on Health and Social Sciences, Portalegre Polytechnic University, Portalegre, 7300-555, Portugal; c Department of Education and Psychology, University of Aveiro, Aveiro, 3810-198, Portugal; d ICBAS, University of Porto, Porto 4050-313, Portugal; e RISE-Health Sciences Research Unit, School of Health Sciences, University of Aveiro, Aveiro, 3810-198, Portugal

**Keywords:** Geriatric nursing, Nursing education, Career choice, Intention, Newly graduated registered nurses

## Abstract

**Background:**

Gerontogeriatric competencies are considered essential for preparing nurses to meet the complex care needs of ageing populations. However, the intention to work with older adults remains comparatively low among nursing professionals, particularly among newly graduated registered nurses (NGRNs).

**Objective:**

This study explored the structural relationships between nursing education, gerontogeriatric competencies, ageism, and the intention to work with older adults.

**Design:**

A nationwide cross-sectional study was conducted.

**Settings and participants:**

This nationwide cross-sectional study was conducted in Portugal and included NGRNs who had completed their undergraduate nursing degree in the 2021–2022 academic year. The final sample comprised 242 NGRNs from public and private nursing education institutions.

**Methods:**

An exploratory structural equation modelling approach was used to examine potential relationships among five latent constructs: Nursing Education (NEDU), Gerontogeriatric Competencies (GGC), Hostile Ageism (AAS_HA), Benevolent Ageism (AAS_BA), and Intention to Work with Older Adults (INT_OA). Model estimation followed an iterative refinement process.

**Results:**

Model estimation encountered convergence difficulties, and the full structural framework could not be fully identified. Consequently, global model fit indices could not be obtained, and the results should be interpreted as exploratory structural associations. Among the estimated paths, a statistically significant association was identified between nursing education and gerontogeriatric competencies (b = 0.437, 95% CI [0.31, 0.57], p < 0.0001), while no statistically significant structural associations were observed between competencies or ageism to the intention to work with older adults.

**Conclusions:**

Nursing education was positively associated with perceived gerontogeriatric competence; however, the intention to work with older adults appears to be shaped by more complex and potentially contextual factors. These findings highlight the need for theoretical refinement and further research using alternative modelling strategies and larger samples.

**What is already known**

• The intention to work with older adults is often low among NGRNs, despite the increasing healthcare needs of ageing populations.
• Nursing education, gerontogeriatric competencies, and ageism have been proposed as factors influencing NGRNs’ intention to work with older adults.
• However, the structural relationships among nursing education, gerontogeriatric competencies, ageism, and NGRNs’ intention to work with older adults remain unclear.

Alt-text: Unlabelled box dummy alt text

**What this paper adds**

• This study explores the structural relationships among nursing education, gerontogeriatric competencies, ageism, and NGRNs’ intention to work with older adults.
• Nursing education was positively associated with NGRNs’ perceived gerontogeriatric competencies.
• Gerontogeriatric competencies and ageism were not statistically significantly associated with NGRNs’ intention to work with older adults, suggesting that career intentions toward older adult care may be shaped by broader contextual and workforce-related influences.

Alt-text: Unlabelled box dummy alt text

## 1 Background

Population ageing is widely recognised as one of the most profound demographic, epidemiological, and clinical transformations of the 21st century. The World Health Organization (WHO) estimates that, by 2050, the global population aged 60 years and older will double, substantially increasing demand for health systems capable of addressing complex, chronic, and multimorbidity-related needs (World Health Organization, 2022). This demographic shift intensifies workforce challenges and reinforces the urgency of preparing and retaining nurses who are both competent and motivated to deliver high-quality gerontogeriatric care across diverse healthcare settings.

Despite this growing need, gerontogeriatric nursing continues to be perceived as a less desirable career pathway across different stages of the nursing profession. Studies among registered nurses (RNs) report limited motivation to work in gerontological settings, often associated with perceptions of lower professional recognition and demanding working conditions (Neville and Dickie, 2014). Similar patterns have been observed among newly graduated registered nurses, whose career preferences frequently favour high-acuity hospital specialties over older adult care (Catalão et al., 2024). Evidence among nursing students also indicates that geriatric care is consistently ranked below high-intensity or technology-oriented specialties, such as critical care or emergency nursing (Kloster et al., 2007; Matarese et al., 2026; Stevens, 2011). These preferences have been linked to perceptions of limited professional prestige, constrained career development opportunities, emotional burden, and insufficient exposure to positive role models in gerontogeriatric care (Neville and Dickie, 2014).

Career motivation towards older adult care appears to be shaped by a combination of personal experiences, professional values, perceived working conditions, and evaluations of the field’s scope and status (Matarese et al., 2026). Within this broader set of influences, attitudinal factors towards ageing and older adults have been identified as particularly relevant in shaping specialty preferences. For example, King et al. (2013) demonstrated that nursing students’ attitudes towards older adults were associated with their preferences for geriatric practice. Similarly, Thuong et al. (2025) found that knowledge and attitudes were related to willingness to care for older adults, highlighting the interplay between educational exposure and attitudinal processes.

These findings are consistent with previous evidence showing persistent specialty preferences among nursing students and newly graduated registered nurses (Kloster et al., 2007; Stevens, 2011). Such trends highlight the need to better understand how educational experiences, competence development, and attitudinal factors may influence professional orientation towards older adult care.

Nursing education is frequently positioned as a central lever for addressing these workforce gaps. However, available evidence suggests that educational approaches and outcomes in gerontogeriatric nursing remain heterogeneous. A scoping review of baccalaureate educational interventions reported substantial variation in programme design, pedagogical strategies (including simulation and e-learning), and outcome measures, limiting the ability to determine which approaches consistently enhance preparedness and sustained interest in older adult care (Baskerville et al., 2025). Recent evidence has also highlighted persistent gaps in nurses’ knowledge of older adult care and emphasised the importance of strengthening gerontological education and continuing professional development to promote more positive attitudes towards caring for older adults (Alhroub et al., 2025). Curriculum development research further emphasises that the integration, visibility, and experiential components of gerontogeriatric content within nursing programmes shape students’ perceived readiness and professional orientation (Tavares et al., 2021). Clinical placements in residential aged care settings, for example, may influence both perceptions of older adult care and subsequent career intentions, underscoring the importance of experiential learning (Abbey et al., 2006). In parallel, the development and validation of gerontogeriatric competency frameworks have advanced the multidimensional assessment of professional competence relevant to clinical performance and identity formation (Catalão et al., 2024).

Nevertheless, educational exposure and competence development alone may be insufficient to fully explain professional orientation towards older adult care. Ageism has been repeatedly identified as a salient attitudinal factor shaping how students perceive older adults and evaluate gerontogeriatric nursing as a career option. Recent evidence also indicates that insufficient knowledge about older adults and persistent ageist behaviours may coexist among nursing students, whereas greater satisfaction with geriatric nursing education and confidence in caring for older adults are associated with more positive attitudes and behaviours, as well as greater knowledge of ageing (Hammad et al., 2025). Among newly graduated registered nurses, gerontogeriatric education and sociodemographic characteristics have been shown to be associated with levels of ageism (Catalão et al., 2025). Furthermore, recent evidence suggests that ageism may exert both direct and indirect effects on career motivation towards gerontogeriatric nursing, operating through mechanisms such as the quality of intergenerational contact and motivational beliefs related to expectancy and value (Chen and Wan, 2025).

These findings suggest that educational, competency-related, and attitudinal factors may operate through interconnected pathways that influence professional intentions towards older adult care. According to the Theory of Planned Behaviour, behavioural intention is influenced by attitudes, subjective norms, and perceived behavioural control (Ajzen, 1991). Within gerontological nursing, attitudes towards older adults and perceptions of geriatric nursing have been identified as important determinants of willingness to pursue careers in older adult care settings (Che et al., 2018; Zheng et al., 2025). Educational experiences, such as gerontological coursework, clinical placements, and structured exposure to older adults, have been associated with greater gerontological knowledge, stronger competencies, and increased willingness to work with older populations (Ho et al., 2023; Liu et al., 2026; Okuyan et al., 2020). Furthermore, evidence suggests that ageism may mediate the relationships among knowledge, educational experiences, and willingness to work with older adults, whereas attitudes towards older adults may mediate the relationship between ageism and professional intentions (Chen and Wan, 2025; Gherman et al., 2022; Pang et al., 2025; Vitman-Schorr and Rozani, 2025). Collectively, these findings support a conceptual framework in which nursing education contributes to the development of gerontogeriatric competencies and age-related attitudes, which, in turn, may influence newly graduated registered nurses’ intentions to work with older adults.

Within behavioural science, intention is widely regarded as the most proximal predictor of behaviour (Ajzen, 1991), and meta-analytic evidence supports a robust association between intention and subsequent action across contexts (Sheeran, 2002). In nursing workforce research, this relationship is particularly relevant, as early career intentions may translate into employment trajectories, retention patterns, and turnover decisions. For example, predictors of actual turnover among NGRNs have been empirically examined, demonstrating that early professional orientations and contextual factors contribute to workforce stability (Brewer et al., 2012). Consequently, intention to work with older adults can be conceptualised as a measurable and theoretically grounded construct reflecting professional orientation and potential career direction.

Taken together, nursing education, gerontogeriatric competence, ageism, and intention-related processes may jointly shape NGRNs’ professional orientation towards older adult care. However, the structural relationships among these variables remain insufficiently clarified, particularly when examined simultaneously within an integrative analytical framework. Empirical testing of these interconnections is therefore warranted.

This exploratory study aimed to understand the factors influencing NGRNs’ intention to work with older adults by analysing the influence of nursing education, gerontogeriatric competencies, and ageism through structural equation modelling (SEM).

### 1.1 Hypotheses

**Fig. 1:**
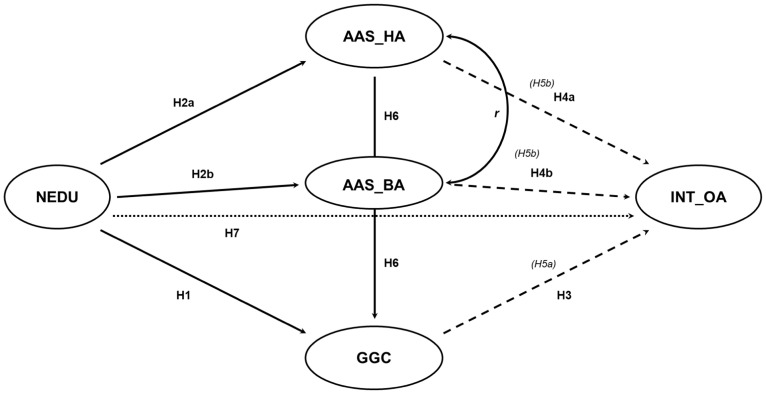
Proposed structural model illustrating the hypothesised relationships.

Note: Solid arrows represent direct paths to mediators and internal effects (H1, H2a, H2b, H6), dashed arrows represent the mediated pathways leading to the outcome (H3, H4a, H4b, H5a, H5b), and the dotted arrow represents the exploratory direct effect of NEDU on INT_OA (H7). Indirect (mediated) effects (H5a, H5b) are implied through the sequential paths connecting Nursing Education to Intention to Work with Older Adults via Gerontogeriatric Competencies and the ageism constructs. The double-headed curved arrow indicates the correlation between the two dimensions of ageism (AAS_HA and AAS_BA). Abbreviations: NEDU = Nursing Education; GGC = Gerontogeriatric Competencies; AAS_HA = Hostile Ageism; AAS_BA = Benevolent Ageism; INT_OA = Intention to Work with Older Adults.

Given the exploratory nature of this study, the proposed relationships were formulated as theory-informed hypotheses derived from prior empirical findings (Fig. 1). These hypotheses were used as guiding assumptions to structure the exploratory SEM analysis rather than as strict confirmatory tests of a fully established theoretical model. Nursing Education (NEDU) was conceptualised as the primary antecedent variable in the model. The following hypotheses were tested:

H1 – Nursing education positively influences gerontogeriatric competencies.
H2a – Nursing education reduces levels of hostile ageism.
H2b – Nursing education reduces levels of benevolent ageism.
H3 – Higher perceived gerontogeriatric competencies positively influences the intention to work with older adults.
H4a – Higher levels of hostile ageism negatively influence the intention to work with older adults.
H4b – Higher levels of benevolent ageism negatively influence the intention to work with older adults.
H5a – Gerontogeriatric competencies may act as a potential mediating pathway linking nursing education and the intention to work with older adults.
H5b – Hostile ageism and benevolent ageism may represent potential mediating pathways linking nursing education and the intention to work with older adults.
H6 – Higher levels of hostile ageism negatively influence the perception of gerontogeriatric competencies.
H7 – Nursing education has a direct effect on the intention to work with older adults.

Fig. 1 visually depicts the proposed relationships between nursing education, gerontogeriatric competencies, hostile and benevolent ageism, and the intention to work with older adults.

## 2 Methods

A nationwide cross-sectional study was carried out within the framework of the project "Gerontogeriatric Competencies of Newly Graduated Nurses." This study is reported in accordance with the STROBE (Strengthening the Reporting of Observational Studies in Epidemiology) guidelines (Vandenbroucke et al., 2007) and followed established recommendations for reporting research using SEM (Schreiber et al., 2006).

Ethical approval was granted by the Health Sciences Research Unit: Nursing (UICISA: E) at the Nursing School of Coimbra (Approval No. 776/04-2021), and all participants provided informed consent prior to participation.

### 2.1 Design and participants

This study was conducted in Portugal and focused on all NGRNs from the 2021–2022 academic year. Participants had completed a four-year undergraduate nursing degree within the previous six months and had obtained professional registration with the Portuguese Order of Nurses. They were recruited from all regions of Portugal, including the autonomous regions, using a non-probabilistic convenience sampling strategy. Responses were excluded if the questionnaire was incomplete or if the respondent was not a registered nurse.

A total of 272 responses were collected. After excluding incomplete submissions, the final analytic sample consisted of 242 NGRNs, corresponding to a completion rate of 88.97%. As the study used a nationwide convenience sampling approach targeting all eligible newly graduated registered nurses, no *a priori* sample size calculation was performed.

To examine the proposed associations among the variables, the study used an exploratory SEM approach. The model was intended to investigate the potential pathways through which nursing education, gerontogeriatric competencies, and ageism might influence the intentions to work with older adults. The model was grounded in theoretical assumptions and prior empirical research, with the goal of identifying key relational patterns in the context of gerontogeriatric nursing workforce development.

### 2.2 Instrument

Data were collected using an online survey composed of seven main sections. To improve clarity and readability, the survey components are reported below according to five thematic domains: sociodemographic characteristics and previous contact with older adults; educational factors and gerontogeriatric learning experiences; ageism towards older adults; gerontogeriatric competencies; and intention to work with older adults.

#### 2.2.1 Sociodemographic characteristics and previous contact with older adults

Sociodemographic variables included age, sex, region of residence, marital status, type of nursing school, living arrangements, and family composition. Additional items assessed participants’ previous contact with older adults, including whether they had lived with an older person, had regular contact with older adults, had previous experience in providing care to older adults outside their family, and maintained regular communication with older adults in family, social, voluntary, academic, or professional contexts.

#### 2.2.2 Educational factors and gerontogeriatric learning experiences

Educational factors related to gerontogeriatric nursing were assessed through items addressing curricular exposure, perceived preparation, clinical training, and learning experiences related to older adult care. As data were collected after participants had completed their undergraduate nursing degree, and no educational intervention was delivered as part of the study, these items assessed participants’ retrospective perceptions of the gerontogeriatric education received during their nursing programmes.

This domain included whether the nursing curriculum explicitly addressed gerontogeriatric competencies, participants’ self-assessed confidence and knowledge in caring for older adults, satisfaction with gerontogeriatric content in nursing education, and perceptions of the competencies and learning outcomes achieved through theoretical instruction and practical experience. It also included items concerning clinical training, such as whether placements were mandatory or elective, the settings in which they took place, the perceived adequacy of training duration, the timing of placements within the programme, the role of supervision and mentorship, and the perceived influence of these experiences on attitudes towards working with older adults.

Curriculum-related items assessed the extent to which participants perceived that their nursing programme prioritised gerontogeriatric content. These items addressed curriculum support for competency development, the qualifications and engagement of teachers responsible for gerontogeriatric content, the distribution of time and academic credits relative to other life stages, and whether clinical training focused mainly on basic care or also included more advanced clinical competencies (Catalão et al., 2025).

#### 2.2.3 Ageism towards older adults

Ageism was assessed using the Ambivalent Ageism Scale (AAS), originally developed by Cary et al. (2017) and subsequently adapted and validated for the Portuguese population by Barroso (2018). The scale comprises 13 items rated on a 7-point Likert scale ranging from 1 (“strongly disagree”) to 7 (“strongly agree”). Higher scores indicate stronger ageist attitudes. The AAS includes two dimensions: Benevolent Ageism (AAS_BA; 9 items) and Hostile Ageism (AAS_HA; 4 items). Evidence from the Portuguese validation study supported the instrument’s reliability and validity, with excellent internal consistency for the total scale (α = 0.90), Benevolent Ageism (α = 0.89), and Hostile Ageism (α = 0.84).

#### 2.2.4 Gerontogeriatric competencies

Gerontogeriatric competencies were assessed using the Gerontogeriatric Competency (GGC) Scale, developed by Tavares et al. (2021) and validated for use with Portuguese newly graduated registered nurses (Catalão et al., 2024). The instrument assesses participants’ self-perceived competencies in caring for older adults and includes 64 items distributed across nine subscales: Communication (six items), Ethics and Deontology (six items), Care for Older Adults (21 items), Safety and Quality (nine items), Family and/or Caregiver Involvement (eight items), Interdisciplinarity (four items), Health Promotion and Disease Prevention (three items), Management (three items), and Professional Development (four items).

Responses were recorded using a 5-point Likert scale ranging from 1 (“Not competent”) to 5 (“Extremely competent”). Higher scores indicated greater self-perceived competence. Total and subscale scores were calculated as the sum of item scores. Confirmatory factor analysis supported the model fit of the nine-subscale structure. Internal consistency was excellent, with a Cronbach’s alpha of 0.992 for the full scale and subscale values ranging from 0.935 to 0.983 (Catalão et al., 2024).

#### 2.2.5 Intention to work with older adults

Intention to work with older adults was assessed through items addressing participants’ career preferences and motivation related to gerontogeriatric nursing. These items explored how strongly participants prioritised working with older adults when ranking preferred nursing specialties, their motivation to choose this population compared with intensive care, and their interest in pursuing a career specifically in gerontogeriatric nursing. These items captured motivational and attitudinal components of career intention and were later used to construct the latent variable representing intention to work with older adults in the SEM.

Three specific indicators were used to operationalise this construct:

• INT_PrefOA: a variable derived from a ranking question in which participants listed their top five preferred nursing specialties. This variable was recoded into a score indicating how highly "older adult care" was ranked (with higher values indicating higher preference; 0 = not selected, 5 = first choice);
• INT_Motiv_OA: a dichotomous item comparing the participant's motivation to work with older adults versus intensive care. Responses were recoded as 1 = preference for older adults, 0 = other;
• INT_Interest_OA: a Likert-scale item measuring the participant’s interest in pursuing a career in gerontogeriatric nursing (1 = not interested at all to 5 = very interested).

These three indicators were selected based on their theoretical relevance and acceptable inter-item correlations. Although they differ in measurement scale, they were retained because they capture complementary dimensions of professional intention, including specialty preference, comparative motivational choice, and explicit career interest. This measurement heterogeneity should be considered when interpreting the results.

### 2.3 Data collection procedures

Data were collected after participants had completed their undergraduate nursing degree and had obtained professional registration with the Portuguese Order of Nurses. No educational intervention was delivered as part of the study. Data collection was carried out using an online questionnaire available between July 2021 and January 2022. Before the survey was launched, permission was obtained from the Portuguese Order of Nurses, which collaborated in the dissemination process. Following approval, the Order emailed the survey link to eligible nurses, together with an information sheet outlining the purpose of the study, the voluntary nature of participation, and assurances of confidentiality. To increase visibility, the survey announcement and access link were also posted on the Order’s official news webpage throughout the data collection period. Additionally, monthly reminder messages were sent to individuals who had not yet completed the questionnaire to encourage participation and improve the overall response rate.

### 2.4 Variables and constructs

This study employed five latent constructs within the SEM framework: NEDU, GGC, AAS_HA, AAS_BA, and INT_OA. The specification and operationalisation of each construct were informed by prior empirical research and psychometric validation studies conducted in earlier phases of the project (Catalão et al., 2024, Catalão et al., 2025).

NEDU was modelled as a first-order latent construct reflecting the overall quality and scope of gerontogeriatric content in undergraduate nursing programmes. The construct integrated indicators capturing three conceptual domains: perceived confidence and preparation, structured curricular exposure to gerontogeriatric competencies, and educational context. One indicator (EDU_StereotypeReinforce) was reverse-coded because its conceptual meaning was opposite to that of the other indicators, such that higher scores reflected lower educational quality. Higher scores therefore indicated more positive educational experiences. Indicators demonstrating low statistical contribution in preliminary analyses were excluded from the final structural model to enhance parsimony and model stability.

GGC was specified as a second-order latent construct derived from nine first-order competency domains of the validated GGC scale. Each domain was represented by the mean of its respective items and used as an indicator of the second-order latent construct in the SEM analysis.

AAS was represented by two correlated first-order latent constructs: AAS_HA and AAS_BA. Each dimension was operationalised using composite mean scores derived from validated scale items. This approach was adopted to represent the overall dimensions of ageism while maintaining model parsimony. As both were modelled as single-indicator latent variables, measurement error was estimated using reliability-adjusted variance. Internal consistency for the ageism dimensions in the present sample was acceptable, with Cronbach’s alpha values of α = 0.850 for AAS_BA and α = 0.882 for AAS_HA. The measurement error variance for each single-indicator latent variable was calculated as Var(x) × (1 − reliability), following standard recommendations for reliability-adjusted single-indicator constructs in SEM (Kline, 2016).

INT_OA was modelled as a latent construct composed of three indicators reflecting preference, motivation, and career interest in gerontogeriatric nursing. These indicators were selected based on their conceptual relevance and were used to operationalise the intention construct in the SEM model.

A detailed description of all observed indicators used to operationalise the latent constructs is provided in Appendix A.

### 2.5 Model specification

The structural model was specified on the basis of the hypotheses presented in Section 1.1 and illustrated in Fig. 1. Each structural path corresponded to a predefined hypothesis concerning the relationships among nursing education, gerontogeriatric competencies, ageism, and the intention to work with older adults.

This model was theoretically grounded and informed by prior empirical findings (Catalão et al., 2024, Catalão et al., 2025), with NEDU specified as the primary exogenous predictor. It represents the initial conceptual framework proposed for the study. However, the final estimated SEM model differed from this initial specification because some hypothesised paths could not be retained during model estimation. The model included five latent constructs: NEDU, GGC, AAS_HA, AAS_BA, and INT_OA.

Direct effects were specified according to the conceptual framework. NEDU was hypothesised to positively predict GGC (H1) and to reduce AAS_HA (H2a) and AAS_BA (H2b). GGC was expected to positively influence INT_OA (H3), whereas AAS_HA and AAS_BA were hypothesised to negatively predict INT_OA (H4a, H4b). An additional exploratory path was specified from AAS_HA to GGC (H6). A direct exploratory effect of NEDU on INT_OA was also included (H7) to assess potential residual effects beyond mediated pathways.

Indirect effects were specified to examine mediation mechanisms. Specifically, the relationship between NEDU and INT_OA was hypothesised to be mediated by GGC (H5a) and by AAS_HA and AAS_BA (H5b).

Regarding measurement structure, NEDU was modelled as a first-order latent construct with indicators reflecting confidence and preparation, structured exposure, and educational context (reverse-coded where appropriate). GGC was specified as a second-order latent construct composed of nine first-order domains. AAS_HA and AAS_BA were modelled as correlated first-order latent constructs represented by composite mean scores. INT_OA was specified as a latent construct composed of three observed indicators (INT_PrefOA, INT_Motiv_OA, and INT_Interest_OA).

Measurement models were evaluated prior to structural estimation, and parameters were estimated using maximum likelihood. However, due to convergence difficulties and partial identification during the iterative estimation process, global fit indices could not be reliably obtained. Weak indicators were therefore excluded to improve model parsimony and stability.

### 2.6 Analysis

Statistical analyses were conducted using IBM SPSS Statistics version 27 (IBM Corp., Armonk, NY, USA) for descriptive statistics. Inferential analyses were conducted using SEM, implemented in IBM SPSS Amos (version 28) and Stata (version 16.0). Given the exploratory nature of the proposed framework and the relatively limited sample size, in relation to model complexity, the SEM analysis was conducted primarily to explore potential structural associations rather than to confirm a fully specified theoretical model (Kline, 2016; Wolf et al., 2013).

Given the complexity of the model, including multiple latent constructs (NEDU, GGC, AAS_HA, AAS_BA, and INT_OA) and several hypothesised paths, an iterative model-building process was necessary. Initially, all hypothesised paths (H1–H7) were included. However, several paths failed to converge or returned non-statistically significant results, requiring stepwise adjustments to the model.

Paths that consistently failed to converge (e.g., NEDU → INT_OA and AAS_HA → GGC) were removed from the final model to improve stability. Consequently, hypotheses H6 and H7 could not be evaluated within the final structural specification. Similarly, non-statistically significant indicators were excluded to enhance model fit and parsimony, including “Mandatory internship in a residential care facility for older adults” (b = −0.031, 95% CI [−0.18, 0.12], p = 0.678), and “Clinical training focused on basic care” (b = 0.04, 95% CI [−0.11, 0.19], p = 0.558). Despite these modifications, the model was only partially identified.

Path coefficients (b) with p < 0.05 were considered statistically significant. The direction of the effect (positive or negative) was interpreted based on the sign of the coefficient.

Model evaluation was planned through the assessment of global goodness-of-fit indices, including the Root Mean Square Error of Approximation (RMSEA), the Comparative Fit Index (CFI), and the Standardised Root Mean Square Residual (SRMR), in accordance with conventional SEM reporting standards. Good model fit was assumed when the ratio of χ^2^ to degrees of freedom was < 3.0, CFI and TLI were ≥ 0.90, SRMR < 0.08, and RMSEA < 0.06, although values between 0.08 and 0.10 were considered acceptable (Byrne, 2016; Hu and Bentler, 1999; Marôco, 2014). However, due to convergence difficulties and partial model identification during the iterative estimation process, reliable global fit indices could not be obtained for the final model specification.

Statistical significance was assessed for direct, indirect, and total effects, with p < 0.05 indicating statistically significant associations.

## 3 Results

### 3.1 Sample characterisation

The sample primarily comprised NGRNs (Table 1), with 83.9% identifying as female and 90.5% as single. The median age was 23 years (IQR = 22–25). Most participants (79.8%) had completed their nursing education at public institutions, and over three-quarters lived in the central (50.4%) or southern (26.4%) regions of Portugal. In terms of family background, 86.4% came from nuclear or single-parent families. When asked about their interactions with older adults, 40.9% reported having lived with an older person, 91.3% had regular contact, and 88.8% had experience in providing care to older adults outside their family. Regular contact was most frequently reported with grandparents or other older relatives (47.5%). Additionally, 97.9% reported maintaining frequent communication with older adults, primarily through paid work (59.5%) and voluntary activities (33.9%).

**Table 1:** Sociodemographic characteristics of the sample (*n* = 242).

	*n* (%)
**Sex**	
Male	39 (16.1%)
Female	203 (83.9%)
**Marital status**	
Single	219 (90.5%)
Married/civil union	23 (9.5%)
**Type of nursing school**	
Private	49 (20.2%)
Public	193 (79.8%)
**Geographical area of residence**	
South	64 (26.4%)
Centre	122 (50.4%)
North	50 (20.7%)
Autonomous region	6 (2.5%)
**Type of family**	
Extended/blended	25 (10.3%)
Nuclear/single-parent	209 (86.4%)
Other	8 (3.3%)
**Lived with older adults**	
Yes	99 (40.9%)
No	143 (59.1%)
**Regular contact with older adults**	
Yes	221 (91.3%)
No	21 (8.7%)
If “yes”: Type of contact	
Grandparents	61 (25.2%)
Parents	10 (4.1%)
Grandparents, other relatives and neighbours	33 (13.6%)
Grandparents, other relatives and other older adult contexts	115 (47.5%)
**Previous experience in caring for older adults (non-relatives)**	
Yes	215 (88.8%)
No	27 (11.2%)
**Regular communication with older adults in a family, social, or professional context**	
Yes	237 (97.9%)
No	5 (2.1%)
If “yes”: Type of context	
Voluntary work	82 (33.9%)
Participation in projects	37 (15.3%)
Paid work	144 (59.5%)
Replacement of the family caregiver	42 (17.4%)
Clinical teaching	30 (12.4%)

### 3.2 Structural equation modelling (SEM)

As previously described, the SEM analysis was conducted through an iterative modelling process aimed at achieving the most stable and interpretable solution. Data from 242 participants were included in the initial specification, and the model was refined through an iterative estimation process aimed at achieving the most stable and interpretable solution.

The path from nursing education to gerontogeriatric competencies was statistically significant (b = 0.437, 95% CI [0.31, 0.57], p < 0.0001), supporting H1, which proposed that nursing education positively predicted gerontogeriatric competencies.

In contrast, the influence of nursing education on hostile ageism and benevolent ageism was not statistically significant (p > 0.05; see Table 2).

**Table 2:** Structural paths among latent variables (*n* = 242).

Hypothesis	Relationship	*b*	SE	z	*p*	95% CI
H2a	NEDU → AAS_HA	0.109	2.218	0.05	0.961	(−4.23; 4.46)
H2b	NEDU → AAS_BA	0.381	0.673	0.57	0.572	(−0.94; 1.70)
H1	NEDU → GGC	0.437	0.065	6.67	**< 0.0001**	(0.31; 0.57)
H4a	AAS_HA → INT_OA	−0.173	3.608	−0.05	0.962	(−7.25; 6.90)
H4b	AAS_BA → INT_OA	0.570	1.251	0.46	0.648	(−1.88; 3.02)
H3	GGC → INT_OA	−0.043	0.111	−0.39	0.698	(−0.26; 0.17)

NEDU = Nursing Education; GGC = Gerontogeriatric Competencies; AAS_HA = Hostile Ageism; AAS_BA = Benevolent Ageism; INT_OA = Intention to Work with Older Adults; b = unstandardised regression coefficient; SE = standard error; CI = confidence interval. H5a and H5b refer to indirect (mediated) effects and are therefore not presented among the direct structural paths shown in this table.

Similarly, no statistically significant association was observed between gerontogeriatric competencies and the intention to work with older adults, providing no support for H3 (p > 0.05).

Likewise, neither hostile ageism nor benevolent ageism significantly predicted the intention to work with older adults (p > 0.05). Hypotheses H5a and H5b proposed indirect pathways linking nursing education to intention through gerontogeriatric competencies and ageism. However, given the absence of statistically significant associations among the relevant structural paths, no evidence was found to support these proposed mediating relationships.

Finally, two hypothesised paths (H6 and H7) were not retained in the final model due to convergence problems during estimation. Table 4 summarises the status of each study hypothesis, distinguishing between hypotheses that were tested, supported, not supported, or not evaluated in the final model.

To ensure construct validity, the measurement models for each latent variable were evaluated individually. The measurement model for NEDU was initially estimated including all theoretically relevant indicators. Table 3 presents the factor loadings obtained for this initial specification. However, two items were found to be non-statistically significant and were excluded from the final structural specification: “Mandatory internship in a residential care facility for older adults” (b = −0.031, 95% CI [−0.18, 0.12], p = 0.678) and “Clinical training focused on the development of basic care” (b = 0.04, 95% CI [−0.11, 0.19], p = 0.558).

**Table 3:** Factor loadings for the observed indicators of nursing education (NEDU) in the initial measurement model.

*Indicator*	*b*	SE	z	*p*	95% CI
CONF_Knowledge_OA	0.449	0.063	7.06	**< 0.0001**	(0.32; 0.57)
PREP_Perceived_OA	0.53	0.059	9	**< 0.0001**	(0.42; 0.65)
TRAIN_Adequacy_Weeks	0.518	0.06	8.6	**< 0.0001**	(0.40; 0.64)
TRAIN_EndFacilitated	0.369	0.067	5.46	**< 0.0001**	(0.24; 0.50)
EXP_MandatoryInternship	−0.031	0.076	−0.41	0.678	(−0.18; 0.12)
EXP_CurricDefinedComp	0.470	0.063	7.47	**< 0.0001**	(0.35; 0.59)
EXP_CurricPromotedComp	0.73	0.047	15.41	**< 0.0001**	(0.64; 0.82)
EXP_EqualCredits_OA	0.58	0.056	10.32	**< 0.0001**	(0.47; 0.69)
EDU_StereotypeReinforce	−0.22	0.074	−2.97	**0.003**	(−0.36; −0.07)
EDU_BasicCareFocus	0.04	0.076	0.59	0.558	(−0.11; 0.19)

*b* = unstandardised regression coefficient; SE = standard error; CI = 95% confidence interval; EDU_StereotypeReinforce was reverse-coded; therefore, the negative factor loading reflects the reverse scoring of the item rather than an inverse relationship with the latent construct.

**Table 4:** Summary of hypothesis evaluation.

Hypothesis	Structural relationship	Final status	Interpretation
H1	NEDU → GGC	Tested and supported	Nursing education was positively associated with gerontogeriatric competencies.
H2a	NEDU → AAS_HA	Tested but not supported	No statistically significant association was found.
H2b	NEDU → AAS_BA	Tested but not supported	No statistically significant association was found.
H3	GGC → INT_OA	Tested but not supported	No statistically significant association was found.
H4a	AAS_HA → INT_OA	Tested but not supported	No statistically significant association was found.
H4b	AAS_BA → INT_OA	Tested but not supported	No statistically significant association was found.
H5a	NEDU → GGC → INT_OA	Not supported	No evidence was found to support the proposed mediating relationship.
H5b	NEDU → AAS_HA/AAS_BA → INT_OA	Not supported	No evidence was found to support the proposed mediating relationship.
H6	AAS_HA → GGC	Not evaluated	Removed due to convergence problems.
H7	NEDU → INT_OA	Not evaluated	Removed due to convergence problems.

NEDU = Nursing Education; GGC = Gerontogeriatric Competencies; AAS_HA = Hostile Ageism; AAS_BA = Benevolent Ageism; INT_OA = Intention to Work with Older Adults.

For the GGC construct, all nine observed indicators demonstrated strong and statistically significant loadings (Table 5), supporting the robustness of the second-order latent variable.

**Table 5:** Factor loadings for observed indicators of gerontogeriatric competencies (GGC) and intention to work with older adults (INT_OA).

*Domain*	Factor loadings of GGC				
	*b*	SE	z	*p*	95% CI
Communication	0.800	0.025	31.92	**< 0.0001**	(0.75; 0.85)
Ethics/Deontology	0.723	0.033	22.18	**< 0.0001**	(0.66; 0.79)
Care for Older Adults	0.897	0.014	62.97	**< 0.0001**	(0.86; 0.93)
Safety and Quality	0.919	0.012	79.69	**< 0.0001**	(0.90; 0.94)
Family and Caregiver Involvement	0.941	0.009	110.58	**< 0.0001**	(0.92; 0.96)
Interdisciplinarity	0.942	0.008	110.83	**< 0.0001**	(0.93; 0.96)
Health Promotion and Disease Prevention	0.935	0.009	100.99	**< 0.0001**	(0.92; 0.95)
Management	0.919	0.011	82.22	**< 0.0001**	(0.90; 0.94)
Professional Development	0.938	0.009	105.47	**< 0.0001**	(0.91; 0.96)
*Indicator*	Factor loadings of INT_OA				
	*b*	SE	z	*p*	95% CI
INT_PrefOA	0.504	0.131	3.84	**< 0.0001**	(0.25; 0.76)
INT_Motiv_OA	0.518	0.104	4.98	**< 0.0001**	(0.31; 0.72)
INT_Interest_OA	0.478	0.109	4.39	**< 0.0001**	(0.26; 0.69)

*b* = unstandardised regression coefficient; SE = standard error; CI = 95% confidence interval.

For the INT_OA construct, all three selected indicators showed statistically significant positive loadings on the latent factor, as shown in Table 5.

For the ageism constructs (AAS_HA and AAS_BA), each was represented by a single composite indicator, and factor loadings were therefore not estimated. As these constructs were modelled as single-indicator latent variables, their parameters were estimated with limited measurement information, which may have contributed to larger standard errors and reduced stability of the associated structural paths. They were modelled separately to examine their unique predictive effects on the intention to work with older adults, in line with the study’s conceptual framework.

## 4 Discussion

This exploratory study examined the structural relationships among nursing education (NEDU), gerontogeriatric competencies (GGC), ageism (AAS_HA and AAS_BA), and the intention to work with older adults (INT_OA) using SEM. Among the examined pathways, a statistically significant association was identified between NEDU and GGC. This finding is consistent with the view that educational experiences are associated with perceived professional competence in gerontogeriatric care.

Although the proposed framework was theoretically grounded and supported by prior empirical research, a fully usable structural model could not be established. Among the hypothesised paths, only the association between NEDU and GGC (H1) was statistically supported. All remaining direct and indirect effects were non- statistically significant, and repeated convergence difficulties prevented the establishment of a stable and well-fitting structural solution.

Support of H1 aligns with existing evidence indicating that structured curricular exposure contributes to higher self-perceived competence in gerontogeriatric care (Catalão et al., 2024, Catalão et al., 2025). This finding suggests the importance of undergraduate education in relation to professional confidence and perceived readiness to care for older adults (Dahlke et al., 2024; Keeping-Burke et al., 2020). The robustness of the GGC measurement model, evidenced by strong and statistically significant loadings across all competency domains, further strengthens this interpretation, suggesting that the competence construct was both psychometrically sound and theoretically coherent in this sample.

By contrast, the absence of statistically significant paths from competencies and ageism to intention indicates that, in this sample, these constructs do not explain intention via the specified direct effects or the hypothesised linear mediation structure. This divergence from previous findings may reflect differences in study populations, contextual settings, and the operationalisation of key variables. For example, Che et al. (2018) examined nursing students using constructs derived from the Theory of Planned Behaviour, in which attitudes, subjective norms, and perceived behavioural control were directly associated with intention. Similarly, Chen and Wan (2025) conceptualised career motivation towards gerontogeriatric nursing through psychological dimensions such as expectancy and value, while also incorporating relational variables including empathy and intergenerational contact.

In contrast, the present study focused on NGRNs transitioning to professional practice and operationalised intention through indicators reflecting specialty preference, comparative motivation, and career interest. Although these indicators capture relevant aspects of professional orientation, they may represent motivational dimensions that differ from those assessed in previous studies. Moreover, differences in cultural context, educational systems, and career stage may influence how competencies and attitudes translate into professional intention. In this context, career intentions among NGRNs may be more strongly shaped by structural and contextual factors, such as employment opportunities, working conditions, or personal circumstances, than by gerontogeriatric competencies or ageism alone. Previous workforce research has shown that employment conditions, career progression opportunities, and remuneration are among the key determinants influencing specialty choice and retention among newly graduated nurses (Brewer et al., 2012; Hayes et al., 2012). This interpretation is consistent with more recent evidence indicating that career intentions towards older adult care are influenced by a complex combination of professional, educational, and contextual factors (Cheng et al., 2022; Chen and Wan, 2025; Matarese et al., 2026). These findings suggest that structural factors may play an important role in early career decisions, alongside educational and attitudinal variables. Shehadeh et al. (2025) highlighted that improving older adult care requires not only educational preparation but also adequate facilities, resources, professional training, and intersectoral collaboration. Together, these considerations raise the possibility that the relationships between gerontogeriatric competencies, ageism, and intention may be more context-dependent than previously assumed and that additional mediating or moderating variables should be considered in future models.

Despite extensive iterative refinement, a fully identified and stable model could not be achieved. Convergence difficulties and partial identification limited the calculation of reliable global fit indices.

The complexity of the proposed SEM relative to the available sample size likely contributed to these estimation difficulties. Additionally, the use of heterogeneous measurement scales for the INT_OA indicators, including a binary indicator and a recoded ranking variable, may have further affected the stability of maximum likelihood estimation. Simulation studies suggest that models including second-order constructs often require substantially larger samples to achieve stable estimation (Wolf et al., 2013). Consequently, the present findings should be interpreted as exploratory structural associations rather than confirmatory evidence of a fully specified theoretical model.

The relatively large standard errors observed in some structural paths involving the ageism constructs may also reflect the modelling of these variables as single-indicator latent constructs, which provide limited measurement information and may reduce estimation stability when combined with modest variability in the constructs.

Although several non-statistically significant paths and weak indicators were removed to improve parsimony, the overall structural solution remained unstable. This outcome highlights the methodological challenges of modelling career intention as a multifactorial construct influenced by educational, attitudinal, contextual, and potentially labour-market–related determinants (Cheng et al., 2022). This pattern aligns with recent mixed-methods evidence showing that motivation to work with older adults is shaped by a complex set of personal, educational, and contextual factors. It also suggests that perceived competence and ageism may operate indirectly or interact with other variables rather than functioning as straightforward direct predictors (Matarese et al., 2026).

Several factors may help explain these results. First, the relative homogeneity of the sample may have constrained variability across several core constructs. Descriptive statistics also indicated relatively high scores across the GGC domains, suggesting that self-perceived competence may have approached the upper end of the scale for many participants. Such patterns may reflect a potential ceiling effect, which can reduce variability and weaken the covariance structure required to detect structural associations. In addition, participants were NGRNs from the same academic cohort, with similar ages and educational backgrounds within a single national system. Such demographic and educational similarity may have reduced dispersion in perceived competence, ageism, and intention scores, thereby limiting the covariance structure necessary for stable SEM estimation. Structural equation modelling relies on sufficient variability in observed indicators to detect latent relationships; restricted variance may attenuate structural effects even when theoretical associations exist (Kline, 2016; Schmidt and Hunter, 2015).

Second, although the overall sample size was acceptable for descriptive analyses, it may have been insufficient relative to the complexity of the proposed model. The inclusion of multiple latent variables, a second-order construct, and several direct and mediated pathways increased the number of estimated parameters. SEM is highly sensitive to model complexity, and the combination of sample size, homogeneity, and structural specification may have compromised estimation stability (Kline, 2016; Wolf et al., 2013).

Beyond methodological considerations, the findings also raise conceptual questions. The absence of statistically significant structural effects does not necessarily imply that competence, education, and ageism are unrelated to professional orientation or intentions. Rather, it may indicate that the directional pathways were overly simplified or that intention to work with older adults is shaped by broader contextual determinants, such as employment opportunities, perceived career progression, institutional culture, remuneration, or work-life expectations (Hayes et al., 2012; World Health Organization, 2020). It is also plausible that competence and ageism exert more distal, interactive, or longitudinal effects that cannot be adequately captured within a cross-sectional structural framework.

This research supports a more refined and realistic understanding of professional intention in gerontogeriatric nursing and provides a basis for revising conceptual frameworks and simplifying structural assumptions. Future studies should adopt more parsimonious models, use longitudinal designs to examine intention-behaviour transitions, and incorporate contextual workforce factors that may mediate or moderate professional orientation. Larger and more heterogeneous samples would likely increase variability and improve structural estimation. Qualitative or mixed-methods approaches may also clarify the mechanisms underpinning career decisions among NGRNs.

The findings of this study have important implications for both nursing education and workforce development. The positive association between nursing education and gerontogeriatric competencies reinforces the need to maintain and strengthen gerontogeriatric content within undergraduate nursing curricula. At the same time, the absence of statistically significant associations among competencies, ageism, and the intention to work with older adults suggests that educational strategies alone may be insufficient to promote career interest in gerontogeriatric nursing. Nursing educators should therefore complement theoretical content with high-quality clinical placements in diverse older adult care settings, intergenerational learning experiences, and pedagogical approaches explicitly designed to address ageist stereotypes and promote positive attitudes towards ageing. Healthcare organisations, educators, and policymakers should consider broader contextual factors, including working conditions, career development opportunities, and professional recognition, when developing strategies to attract newly graduated registered nurses to older adult care settings.

### 4.1 Strengths and limitations

A key strength of this study lies in its integrative examination of educational, competence-related, and attitudinal constructs within a single structural framework. Measurement rigour was further reinforced using validated instruments, including a second-order competency model with robust psychometric properties.

Nevertheless, some limitations should be acknowledged when interpreting these findings. First, the cross-sectional design precludes causal inference. Additionally, the study relied on self-report measures; therefore, gerontogeriatric competencies reflected perceived rather than observed competence, and intention to work with older adults reflected stated intentions rather than actual career behaviour. Second, the reliance on non-probabilistic sampling within a single national context limits the generalisability of the results beyond Portugal. Third, the relative homogeneity of the sample may have restricted variability in key constructs, thereby reducing the sensitivity of the structural equation modelling analysis to detect meaningful associations. Finally, the complexity of the proposed model relative to the available sample size may have compromised estimation stability and hindered model convergence.

## 5 Conclusion

This exploratory study examined the structural relationships between nursing education, gerontogeriatric competencies, ageism, and intention to work with older adults among NGRNs. The findings demonstrated a statistically significant positive association between nursing education and perceived gerontogeriatric competence, suggesting the importance of undergraduate education in strengthening nurses’ preparedness to care for older adults. However, no statistically significant structural pathways were identified linking competencies or ageism to career intention. This suggests that professional decision-making regarding work with older adults may be influenced by additional contextual and motivational factors not captured in the proposed framework.

Given the exploratory nature of the structural modelling and the complexity of the proposed framework relative to the sample size available, these findings should be interpreted with caution and primarily as preliminary structural evidence.

By identifying the limitations of the proposed framework, this study contributes to theoretical refinement and provides direction for future research aimed at strengthening nurses’ professional development in gerontogeriatric care.

## Ethics approval

The study was conducted in accordance with the Declaration of Helsinki and the research protocol and the consent form were approved by the Ethics Committee of the Health Sciences Research Unit: Nursing (UICISA: E) at the Nursing School of Coimbra (Order No. 776/04–2021).

## Informed consent statement

Informed consent was obtained from all subjects involved in the study.

## Funding

This research was funded by Fundação para a Ciência e a Tecnologia, I.P. (grant UID/06173/2025 – CARE - https://doi.org/10.54499/UID/06173/2025).

## CRediT authorship contribution statement

**Maria José Catalão:** Writing – review & editing, Writing – original draft, Visualization, Resources, Methodology, Funding acquisition, Formal analysis, Data curation, Conceptualization. **Helena Arco:** Writing – review & editing, Writing – original draft, Visualization, Supervision, Resources, Data curation. **Nuno Carrajola:** Visualization, Resources, Data curation. **João Tavares:** Writing – review & editing, Writing – original draft, Visualization, Supervision, Resources, Formal analysis, Data curation, Conceptualization.

## Declaration of competing interest

The authors declare that they have no known competing financial interests or personal relationships that could have appeared to influence the work reported in this paper.

## Appendix Supplementary materials

Image, application 1
