# Supplementary material for: Understanding nursing graduates’ intention to work with older adults: An exploratory structural framework
Source: Int J Nurs Stud Adv. 2026 Jul 12;11:100628. doi: 10.1016/j.ijnsa.2026.100628 (PMC13418224; doi:10.1016/j.ijnsa.2026.100628)
Supplement: Supplementary file 1 [file mmc1.docx]

| **Construct** | **Indicator** | **Description** | **Status in final model** |
| --- | --- | --- | --- |
| **NEDU** | CONF_Knowledge_OA | Self-perceived knowledge and confidence | Included |
|  | PREP_Perceived_OA | Perceived preparedness | Included |
|  | TRAIN_Adequacy_Weeks | Adequacy of training duration | Included |
|  | TRAIN_EndFacilitated | End-of-programme placement facilitated learning | Included |
|  | EXP_MandatoryInternship | Mandatory LTC placement | Excluded |
|  | EXP_CurricDefinedComp | Curriculum-defined competencies | Included |
|  | EXP_CurricPromotedComp | Curriculum promoted competencies | Included |
|  | EXP_EqualCredits_OA | Equal academic weighting | Included |
|  | EDU_StereotypeReinforce | Stereotype-reinforcing supervision (reverse-coded) | Included |
|  | EDU_BasicCareFocus | Sole focus on basic care | Excluded |
| **GGC** | Nine subdomains | Second-order construct based on validated GGC scale | Included |
| **AAS_HA** | Composite score | Mean of four hostile ageism items | Included |
| **AAS_BA** | Composite score | Mean of nine benevolent ageism items | Included |
| **INT_OA** | INT_PrefOA | Preference ranking | Included |
|  | INT_Motiv_OA | Motivation comparison | Included |
|  | INT_Interest_OA | Career interest | Included |

**Appendix A**. Operationalisation of latent constructs and observed indicators.

Note. NEDU = Nursing Education; GGC = Gerontogeriatric Competencies; AAS_HA = Hostile Ageism; AAS_BA = Benevolent Ageism; INT_OA = Intention to Work with Older Adults; LTC = Long-Term Care. NEDU was modelled as a first-order latent construct composed of indicators reflecting confidence and preparation, structured curricular exposure, and educational context (reverse-coded where appropriate). GGC was specified as a second-order latent construct based on nine validated competency domains. AAS_HA and AAS_BA were modelled as single-indicator latent variables using composite mean scores, with measurement error estimated based on reliability-adjusted variance. INT_OA was modelled as a latent construct composed of three observed indicators. Indicators marked as “Excluded” were removed from the final structural model due to non-significant factor loadings or instability during model estimation.
